# Cases of Gonorrhæa Successfully Treated by Cubebs

**Published:** 1823-12

**Authors:** Miles Marley

**Affiliations:** Member of the Royal College of Surgeons.


ART. VI.—*Cases of Gonorrhœa successfully treated by Cubebs,* (continued.) By MILES MARLEY, Esq. Member of the Royal College of Surgeons.

IN the month of June, 1821, I published some observations on the efficacy of cubebs in gonorrhœa, with a promise to renew the subject when I had given the medicine a more extended trial; and, having done so, I am anxious to lay the result before the profession. I am aware of the contrariety of opinion that exists respecting the effects of cubebs, and on that account I am the more desirous of giving publicity to the continued success of my practice. By advancing opinions so contrary to those held out by many men so deservedly distinguished in our profession, I, with feelings of diffidence, perform a duty almost necessarily imposed on me. I then gave the result of my experience in a few cases; but having had, since that time, an opportunity of giving the medicine a more extended trial, I can now, with still greater confidence, speak of its good effects. It puzzles me to think why there are so many opposite opinions respecting the effects of any one medicine; for I can fairly and conscientiously say that I have, almost invariably, met with success in those cases where the medicine was applicable,—namely, those of a recent nature.

I have occasionally met with cases where the medicine seemed to have no influence whatever, and I think I may here mention that, if sensible effects are not produced by the time a couple or three ounces have been taken, we had better entirely discontinue it. We may lay it down as a general rule, that a case of above a month's standing will seldom or never yield to the use of cubebs.

Respecting the dose, we should never give less than an ounce in the course of twelve hours; at the same time, the patient should strictly adhere to the antiphlogistic regimen. We are all aware that there is great difficulty in persuading patients to follow the rules laid down for them; and, to their non-compliance on this point, we may sometimes attribute the failure of many valuable remedies, as well as the medicine under consideration. Sometimes (no matter in what form we exhibit the medicine,) the stomach will be found to reject it: in such cases, I have found five or six drops of the tincture of opium of use in allaying the irritability of that organ. The bowels are sometimes constipated, and, when this is the case, the head, as we might expect, generally sympathises: a brisk purge is here indicated, and will be found to give the patient relief. In cases not of a very recent nature, I have found it a good plan to let a copious evacuation precede its exhibition, which may be obtained by three or four grains of calomel at bed-time, and a neutral salt in solution the following morning. I must here remark, that it is absolutely necessary to continue the medicine, with the same restrictions, for three days at least after the discharge has subsided.

It would be useless for me to dwell any longer on the subject; therefore I shall conclude by saying, that I hope and trust the warmth with which I have recommended the medicine will not be construed into any other wish than that of a laudable desire to see introduced into practice, more generally, a remedy that, in one of the most troublesome and frequent complaints, possesses the most specific effects.

I have, within the last few days, been speaking upon the subject with a surgeon of high character and considerable eminence, who generally prescribes it with the most favourable results.

I shall now proceed to transcribe a few of those cases that have lately fallen under my observation.

Case I.—A man, aged about thirty years, became infected four or five days ago; perceived a discharge of thick yellowish matter yesterday morning; now complains of a slight scalding on making water.

June 17th.—Take of cubebs in powder ʒiij. three times a-day, in a little milk.

18th.—Symptoms not increased. Continue.

19th.—No pain on making water; the discharge not much altered in quantity, but thicker in consistence. Continue.

21st.—Apparently free from the disease. Continue three days longer.

Case II.—A gentleman's coachman, aged about thirty-seven years, had connexion three days ago; perceived a discharge this morning; complains of a painful pricking heat, on passing his water.

June 19th.—Take of cubebs in powder ʒiij. three times a-day. The disease in this case was totally eradicated in twenty-four hours. Continue three days longer.

Case III.—A young man became affected about three weeks since: a discharge of a straw-coloured matter was perceived about a week after, with a scalding on making water; has been troubled with chordee for the last night or two. This man has never had the disease before.

July 4th.—Let him take four grains of calomel at bed-time, followed by a purgative draught in the morning.

5th.—The medicine has operated well. Take of cubebs in powder ʒiij. three times a-day in water.

6th.—No improvement; says the medicine produces constant nausea.

7th.—Let him take the cubebs in coffee. The symptoms are materially alleviated. Continue.

9th.—The disease has totally disappeared. Continue four days longer.

Case IV.—Mr. —— has had a profuse discharge of thin matter from the urethra for the last four days, which came on soon after connexion. He this morning, for the first time, complained of pain on making water.

July 6th.—Take of cubebs in powder ʒiij. three times a-day.

7th.—The discharge increased in quantity. Continue.

9th.—The discharge lessened, and the ardor urinæ considerably relieved. Continue.

The discharge nearly gone. Continue the use of the cubebs a week longer.

I do not know the result of the above case, as the patient was unexpectedly called out of town; but I think there can be little doubt of its favourable termination.

CASE V.—A young woman, about twenty years of age, applied to me, with a discharge of matter from the vagina, which she had had for the last fortnight. Two days since, she felt excessive pain on making water, which seems to increase in violence.

July 12th.—Let her take powdered cubebs ʒiij. three times a-day.

13th.—No alteration. Continue.

14th.—The ardor urinæ considerably diminished, the discharge unaltered. Continue.

15th.—She complains of nothing this morning but a slight discharge.

17th.—Cured. Continue three days longer.

CASE VI.—A military man, aged forty-seven years, had connexion some days since: this morning perceived a discharge of purulent matter, with a scalding on making water; was kept awake last night with chordee.

July 15th.—Take of cubebs in powder ʒiij. three times a-day.

16th.—The discharge rather less; chordee very troublesome last night. Let him take a grain of opium at bed-time, and continue the cubebs as before.

17th.—Has had a good night, and is much better.

18th.—The discharge has nearly disappeared. Continue the cubebs.

My patient is quite well, and tells me that he has had the disease several times, and never was cured under a month or six weeks. Continue three days.

CASE VII.—A young gentleman, a medical pupil, had connexion about a fortnight since; the discharge appeared about six days after, and has continued to increase. He has tried the usual remedies without success.

Sept. 8th.—Take of cubebs powdered ʒiij. three times a-day.

10th.—The discharge thicker. Continue.

13th.—Quite well, and free from all symptoms of the disease. Continue three days longer.

13, *Vigo-lane; Oct. 13th*, 1823.

